# Forecasting leading industry stock prices based on a hybrid time-series forecast model

**DOI:** 10.1371/journal.pone.0209922

**Published:** 2018-12-31

**Authors:** Ming-Chi Tsai, Ching-Hsue Cheng, Meei-Ing Tsai, Huei-Yuan Shiu

**Affiliations:** 1 Department of Business Administration, I-Shou University, Dashu District, Kaohsiung City, Taiwan; 2 Department of Information Management, National Yunlin University of Science and Technology, Douliou, Yunlin, Taiwan; Universita Cattolica del Sacro Cuore, ITALY

## Abstract

Many different time-series methods have been widely used in forecast stock prices for earning a profit. However, there are still some problems in the previous time series models. To overcome the problems, this paper proposes a hybrid time-series model based on a feature selection method for forecasting the leading industry stock prices. In the proposed model, stepwise regression is first adopted, and multivariate adaptive regression splines and kernel ridge regression are then used to select the key features. Second, this study constructs the forecasting model by a genetic algorithm to optimize the parameters of support vector regression. To evaluate the forecasting performance of the proposed models, this study collects five leading enterprise datasets in different industries from 2003 to 2012. The collected stock prices are employed to verify the proposed model under accuracy. The results show that proposed model is better accuracy than the other listed models, and provide persuasive investment guidance to investors.

## Introduction

The prices forecast of stock is the most key issue for investors in the stock market, because the trends of stock prices are nonlinear and nonstationary time-series data, which makes forecasting stock prices a challenging and difficult task in the financial market. Conventional time series models have been used to forecast stock prices, and many researchers are still devoted to the development and improvement of time-series forecasting models. The most well-known conventional time series forecasting approach is autoregressive integrated moving average (ARIMA)[1], which is employed when the time-series data is linear and there are no missing values [2]. Statistical methods, such as traditional time series models, usually address linear forecasting models and variables must obey statistical normal distribution [3]. Therefore, conventional time series methods are not suitable for forecasting stock prices, because stock price fluctuation is usually nonlinear and nonstationary.

Further, most conventional time-series models utilize one variable (the previous day’s stock price) only [4], when, there are actually many influential factors, such as market indexes, technical indicators, economics, political environments, investor psychology, and the fundamental financial analysis of companies that can influence forecasting performance [5]. In practice, researchers use many technical indicators as independent variables for forecasting stock prices. How to select the key variables from numerous technical indicators is a critical step in the forecasting process. Investors usually prefer to select technical indicators depending on their experience or feelings for forecasting stock prices despite this behavior be highly risky. However, choosing unrepresentative indicators may result in losing profits for investors. Therefore, selecting the relevant indicators to forecast stock prices is one of the important issues for investors. Financial researchers must identify the key technical indicators that have higher relevance to the stock price by indicator selection. Therefore, proposed models must incorporate indicator selection in the stock forecasting process to enhance forecasting accuracy.

Recently, there have been many new forecasting techniques used to construct efficient and precise machine learning models, but forecasting stock prices is still a hot topic [6, 7, 8]. To overcome the shortcomings of traditional time series models, nonlinear approaches have been proposed, such as fuzzy neural networks [9, 10, 11, 12], and support vector regression (SVR) [13, 14, 15, 16]. SVR utilizes the minimized structural risk principle to evaluate a function by using the minimized the upper bound of the generalized error [17, 18]. The minimized structural risk principle could get better generalization from limited size datasets [19]. Further, SVR has a global optimum and exhibits better prediction accuracy due to its implementation of the structural risk minimization principle, which considers both the training error, and the capacity of the regression model [15, 20]. Although SVR has shown a great number of experimental results in many applications such as economic and financial predictions, the main problem of SVR is the determination of its parameters, which requires practitioner experience [21]. In the literature, genetic algorithms (GA) have been successfully used in a wide range of problems included machine learning, multiobjective optimization problems and multimodal function optimization [22]. GA is a search algorithm inspired by evolution and is usually used to solve optimization problems. Therefore, proposed models utilize GA to optimize the parameters of SVR and obtain better forecasting performance.

From the related work mentioned above, previous studies have shown some drawbacks:

(1) Many researches select key technical indicators depending on experiences and ideas [23], (2) Most statistical methods follow some assumptions in different datasets, and obey the statistical distributions [3], (3) Most previous time series models consider only one feature to forecast stock indexes [23], and (4) The parameter of SVR is difficult to determine [24, 25, 26].

This paper proposes a novel GA-SVR time series model based on indicator selection to overcome these problems, and the proposed model contributes the following: (1) In feature selection, this study applies multivariate adaptive regression spline (MARS), stepwise regression (SR), and kernel ridge regression to get the key technical indicators for investors. (2) The proposed model optimizes the parameters of SVR by genetic algorithm (GA) to increase the forecast accuracy. (3) The results could provide persuasive investment guidelines for investors.

The remaining contents of this paper are organized as follows. Section 2 describes the related methodology that incorporate the technical indicator, MARS, genetic algorithm, SVR, and stepwise regression. Section 3 presents the proposed algorithm. Section 4 provides the experimental results and comparisons. Conclusion of this paper are explained in Section 5.

## Related work

This section introduces the related work containing the technical indicator, multivariate adaptive regression splines, genetic algorithm, support vector regression, and briefly stepwise regression.

### Technical indicator

The technical indicator (TI) is an investment guidance for investors based on evaluating the profits of securities from analyzing trading data of marketing activities, such as past prices and volumes [5]. Stock market data have highly nonlinear, and many researches have focused on the technical indicator to increase the investment return [27, 28]. A technical indicator is a formula, which transfers trading data (open price, the lowest price, the highest price, average price, closing price and volume) into different technical indicators, and try to forecast future prices based on analyzing the past pattern of stock prices [29, 30]. Technical analysis utilizes basic market data, and assumes that the involved factors are included in the stock exchange information [31]. Based on literature review, this paper collected some technical indicators as Table 1. To consider more features, that affect the stock price and volatility, this paper incorporates the microeconomic features that affect the stock price, and these collected factors are listed in Table 2.

**Table 1:** Technique indicators.

Indicator	Explanation
MA5	$\text{MA}5(5\;\text{days}\;\text{moving}\;\text{average})=\frac{{\text{p}}_{\text{c}}+{\text{p}}_{\text{c}-1}+\ldots +{\text{p}}_{\text{c}-\text{i}}}{5}$, i = 4, and p_c_ is the closing index of the current day [32]
MA10	$\text{MA}10\;(10\;\text{days}\;\text{moving}\;\text{average})=\frac{{\text{p}}_{\text{c}}+{\text{p}}_{\text{c}-1}+\ldots +{\text{p}}_{\text{c}-\text{i}}}{10}$, i = 9, and p_c_ is the closing price of the trading day [32]
5BIAS	The difference between the closing price and MA5, which utilizes the stock price nature of returning back to average price for analyzing the stock trends [31]
10BIAS	The difference between the closing price and MA10, which employs the stock price nature of returning back to average price for analyzing the stock trends [31]
RSI	RSI measures the magnitude of recently gain to recently loss in an trial to determine overbought and oversold conditions of an asset [31]
12PSY	PSY12 (12 days psychological line) = (D_up12_/12) * 100, D_up12_ is the number of days when price is going up within 12 days [23]
10WMS%R	Williams %R is usually drawn by using negative values. For analysis and discussion, ignore the negative symbols. It is the best to wait the security’s price until change direction before placing your trading [31]
MACD	MACD presents the difference between a fast and slow exponential moving average (EMA) for closing prices. Fast is a short-period average, and slow is a long period one [31]
MO1	MO1(t) = price(t) − price(t − n), n = 1 [33]
MO2	MO2(t) = price(t) − price(t − n), n = 2 [33]
Transaction volume	Transaction volume presents a basic yet very important element of market timing strategy. Volume gives clues for the intensity of given price moving [34]
CDP value	Divide the previous price movement into five values and make the intraday trading decision based on the five value [31]
Exponential Moving Average (EMA)	EMA is defined as a linear transformation of time series to a smoother time series by $\tilde{{\text{x}}_{\text{t}}}=\lambda \sum_{\text{K}=0}^{\infty }{\left(1-\lambda \right)}^{\text{k}}{\text{x}}_{\text{t}-\text{k}}$ Where 0<λ≤1 is the timescale. When λ = 1, the EMA is the identity transformation: $\tilde{{\text{x}}_{\text{t}}}={\text{x}}_{\text{t}}$; in contrast, many term x_t−k_ effectively contribute to $\tilde{{\text{x}}_{\text{t}}}$ when λ < 1 [35].
Company-Daily price change	$\text{P}=\frac{{\text{P}}_{\text{c}}-{\text{P}}_{\text{o}}}{{\text{P}}_{\text{o}}}*100\%$, P_c_ is the close price of today and P_o_ is the open price of today [36].
TAIEX-Daily index change	$\text{P}=\frac{{\text{P}}_{\text{cT}}-{\text{P}}_{\text{oT}}}{{\text{P}}_{\text{oT}}}*100\%$, P_cT_ is the close index of today and P_oT_ is the open index of today [36].

**Table 2:** Fundamental indicators.

Indicator	Explanation
TAIEX index	This study considered related market index with macroeconomic, TAIEX index is the indicator of fundamental analysis.
Exchange Rate	Conversion rate of US to NT [37]
Prime / Base rate	Prime rate is an interest rate, which is paid by a borrower (debtor) for the use of money that they borrow from a lender [38]. It has a relationship with macroeconomic and indirect affects the stock market.
The Final Best Ask Quote	Each transaction, the system discloses the quote for the lowest offer price [39], the last transaction every day is collected as indicator.
The Final Best Bid Quote	Each transaction, the system discloses the quote for the highest bid price [39], the last transaction every day is collected as indicator.
Price earnings ratio	It is defined as market price per share divided by annual earnings in per share [4, 40].
PBR	Compare a company’s current market price to its book value [41].
Dividend Yield	$\text{R}=\frac{\text{D}}{\text{P}}$, where D is the most recent full year dividend, P is the current share price. [42]
Return of Investment (ROI)	${\text{R}}_{\text{t}}=\frac{\left({\text{V}}_{\text{f}}-{\text{V}}_{\text{i}}\right)}{{\text{V}}_{\text{i}}}*100\%$, V_f_ is the final value of an investment and V_i_ is the initial value of an investment [43].
Log (ROI)	${\text{R}}_{\text{t}}=ln\;\frac{{\text{V}}_{\text{f}}}{{\text{V}}_{\text{i}}}*100\%$, V_f_ is the final value of an investment and V_i_ is the initial value of an investment [44].
Sale Month	This study considered sale monthly, sales growth rate, sales growth rate and the compared rate sale monthly with previous month would affect the company stock price.
Aggregate Sales Growth Rate	R_year_ = ([R_t_ / R_t-1_]-1)*100(%) R_t_ is the total revenue in t year.
Sales Growth Rate	R_month_ = ([R_y_ / R_y-1_]-1)*100(%) R_y_ is the monthly revenue in y year.
Rate compared sale monthly	R = [(R_m_ − R_m-1_)/ R_m-1_] *100(%) R_m_ is the revenue in m^th^ month.
Demand Savings Deposits	This study considered that the rate of demand savings deposits might be a factor of investment. When the rate is low, investors may be willing to take the risk for investment. [45]

### Multivariate adaptive regression splines

Friedman [46] proposed multivariate adaptive regression splines (MARS), it is a simple nonparametric regression algorithm. The main advantages of MARS is its capacity to grasp the complicated data mapping and patterns of high-dimensional data, and produce more simple, easy interpretation models, and its can perform analysis on feature relative importance. In concept, MARS integrates the piecewise linear regressions into a flexible model for solving the nonlinear and complex problems. MARS establishes the final model in a two-stage procedure: Firstly, the forward stage, many spline basis functions are built, the feature can be continuous, ordinal, or categorical. Secondly, the backward stage removes the redundant spline basis functions, it uses the generalized cross-validation (GCV) criterion [46] to evaluate the performance of model subsets for getting the best subset, the lower *G*_*CV*_ value is better. Moreover, the *G*_*CV*_ is defined as Eq (1).

$$G_{CV}(M)=\frac{1}{N}\sum_{i=1}^{N}\frac{{\left[y_{i}-f_{M}(x_{i})\right]}^{2}}{{\left[1-\frac{C(M)}{N}\right]}^{2}}$$ (1)

where N is the number of data records, *C*(*M*) denotes the penalty cost of a model containing M basis functions, the numerator is the lack of fit on the *M* basis function model *f*_*M*_ (*x*_*i*_), the denominator is the penalty for model complexity *C*(*M*) and *y*_*i*_ denotes the target outputs.

### Genetic algorithm

The genetic algorithm (GA) [47] is to search the global optimum by using inspired natural evolve. Moreover, GA has four operators (inheritance, mutation, selection, and crossover) to evolve repeatedly for obtaining the optimal solution. GA has been applied successfully in economic and financial domain [27, 48]. In specific problem, the GA algorithm encodes a potential solutions into the simple chromosome-like data structure, and applies the re-united operators to preserve critical information [3]. This paper referred the GA steps of Goldberg [49], and reorganized as follows:

Step1: Generate an initial population randomly.
Step2: Evaluate fitness of each chromosome.
Step3: Check the stop criterion.
Step4: Select suitable chromosomes based on the parents’ populations.
Step5: Extend crossover to search a new solution by swapping corresponding to segments of a string representation for the parents.
Step6: Employ mutation randomly to change some of the chosen chromosomes.

### Support vector regression

SVR algorithm is a nonlinear kernel-based regression, which tries to find a regression hyperplane with minimized risk in high dimensional space [16]. Compared to the traditional regression model, it estimates the coefficients by minimizing the square loss, SVR uses the *ε*–insensitivity loss function to obtain its parameters. It can be express as:

$$L_{\varepsilon }\left(f\left(x\right),t\right)=\{\begin{array}{ll} |f\left(x\right)-t|-\varepsilon & \;if\;|f\left(x\right)-t|\geq \varepsilon \\ 0 & otherwise \end{array}$$ (2)

where *t* is the desired (target) outputs, and *ε* defines the region of *ε* -insensitivity, when the predicted value falls into the band area, the loss is zero. Contrarily, if the predicted value falls outside of the band area, the loss is equal to the difference between the predicted value and the margin.

Considering empirical risk and structural risk, the SVR model can use slack variables to construct a minimal quadratic programming problem.

$$\text{Min}:\;\frac{1}{2}{\left\|v\right\|}^{2}+∁\sum_{i=1}^{n}(\xi_{i}+\xi_{i}^{*})$$

$$subject\;to\;\{\begin{array}{l} q_{i}-\left(v\cdot \varphi \left(x_{i}\right)\right)-b\leq \varepsilon +\xi_{i}\\ \left(v\cdot \varphi \left(x_{i}\right)\right)+b-q_{i}\leq \varepsilon +\xi_{i}^{*}\\ \xi_{i},\;\xi_{i}^{*}\geq 0,\;for\;i=1,\cdots ,n \end{array}$$ (3)

The symbols ξ_i_ and $\xi_{\text{i}}^{*}$ are two positive slack variables to calculate the error (qi − f(xi)) from the boundaries of the *ε*–insensitivity zone. $\left(\xi_{\text{i}}+\xi_{\text{i}}^{*}\right)$ denotes the empirical risk, $\frac{1}{2}{\left\|\text{v}\right\|}^{2}$ is the structural risk to prevent over-learning and the lack of applied universality and ∁ denotes the regularization constant for specifying the trade-off between the empirical risk and the regularization terms.

Based on the sequentially modifying coefficient C, band area width *ε*, and kernel function Κ, the optimal parameter can be solved by the Lagrange method [19]. This study based on Vapnik [18] utilized the SVR-based regression function, and it is defined as

$$f(x,\text{z})=\sum_{i=1}^{N}\left(\alpha_{i}-\alpha_{i}^{*}\right)K(x,x_{i})+b$$ (4)

where α_i_ and $\alpha_{\text{i}}^{*}$ are the Lagrangian multipliers that satisfy the equality $\alpha_{\text{i}}\alpha_{\text{i}}^{*}=0$, α_i_ and $\alpha_{\text{i}}^{*}\geq 0$. K(x, x_i_) is the kernel function which represents the inner product 〈φ(x_i_), φ(x)〉. The radial basis function (RBF) has been widely used as the kernel function, and this study utilizes RBF because of its capabilities and simple implementation [50].

$$K(x,x_{i})=\text{exp}\left({-\gamma \|x_{i}-x_{j}\|}^{2}\right)$$ (5)

where γ is the RBF width.

### Stepwise regression

SR is a simple multiple regressions, it establishes a model by adding or removing features based on the statistics of F-test, that is, SR utilized the forward and backward procedures to add or remove features based on F statistics. SR adds the feature to the model if the p-value of variable is less than the given significant level (p < .05), and removes the variable from the model if the p-value of variable is greater than the given significant level [51].

## Proposed model

This study based on previous studies has found some drawbacks in time series forecast: (1) Based on subjective experiences and opinions to select important technical indicators [23]. (2) Previous methods need to follow some assumptions in different datasets, and obey the statistical distributions [3]. (3) Previous time series models consider only one feature to forecast the stock index [23]. (4) The best SVR parameters are difficult to determine [24, 25, 26]. To overcome these problems, this study is based on our conference paper [52] to extend the proposed methods for solving the forecast problems. That is, this paper proposes a GA-SVR time series model based on feature selection to forecast the leading industry stock price. Hence, the proposed model contributes the following. (1) This study utilizes MARS, SR, and KRR to choose the key technical indicators for investors. (2) Use a GA to optimize the SVR parameters for enhancing the forecast accuracy. (3) The results can provide the investment guidance to investors.

**Fig 1:**
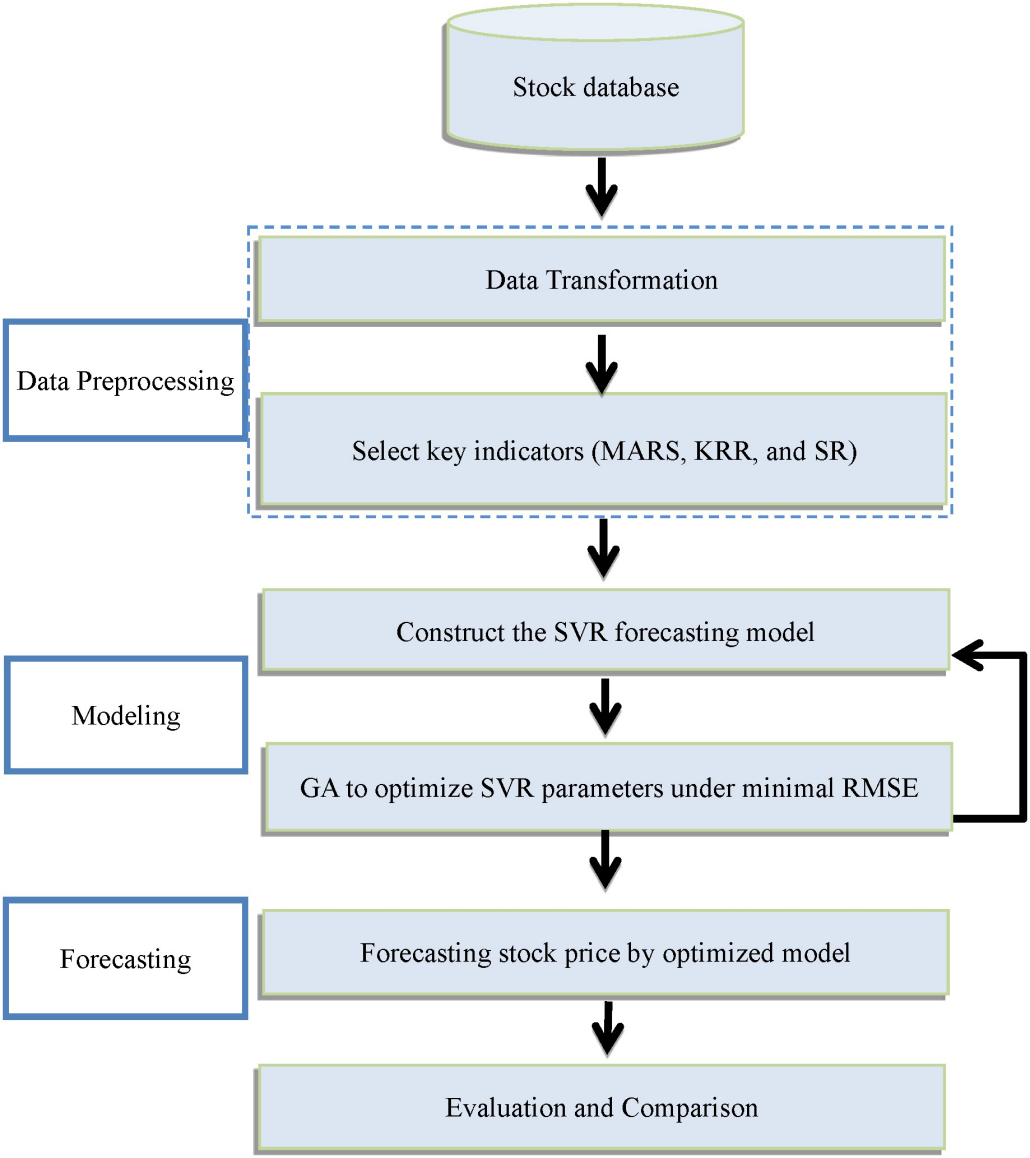
Research processes of the proposed model.

The proposed model included three blocks as Fig 1, it can be briefly described as follows:

1. Data preprocessing: this proposed model transforms daily basic stock data (open price, the lowest price, the highest price, average price, closing price and volume) into technical indicators. Then utilizes MARS, SR, and KRR methods to select the key indicators.
2. Modeling: Build a forecast model by using SVR and employs GA to optimize the parameters of SVR.
3. Forecasting: The optimized GA-SVR forecast model is utilized to forecast the stock price, and compare the proposed models with the listing models under the accuracy.

For easy computation, this section proposed an algorithm with six steps, the detailed step is described as follows:

Step 1: Transform trading data into technical indicators

This step collected daily stock trading data (open, close, the highest, the lowest price and volume), and transformed these data into technical indicators [31], such as MA, PSY, RSI, BIAS, and WMS%R. In addition, this paper also incorporate other indicators, such as exchange rate, NT dollars to US dollars, and the momentum. These technical indicators used are listed in Tables 1 and 2, respectively.

Step 2: Select key features (MARS, SR, and KRR)

From step 1, the collected data has been transformed into technical indicators; this step utilized MARS, SR, and KRR to choose the key indicators. For removing collinearity, this step also run SR multi-collinearity to eliminate the high multi-collinearity indicators. For comparing the three feature selection methods, this study selected the number of features are as similar as possible.

Step3: Construct the SVR forecast model

To build the SVR forecast model, this step used the selected features as input features, and the RBF function is used as the kernel function, due to it can handle the nonlinear and high-dimensional data. To build the forecasting model, three parameters that should be set: the loss function *ε*, the regularization constant *C*, and the RBF width *σ*. To obtain a better forecast model, this step utilizes a genetic algorithm to optimize these parameters.

Step4: Optimize the SVR parameters by GA at minimal RMSE

To get better forecasting accuracy, this step employed genetic algorithm to optimize the SVR parameters *C* and *σ* under minimal RMSE (Eq 6) for training dataset. The RMSE is defined as:

$$\text{RMSE}=\sqrt{\frac{1}{n}\sum_{t=1}^{n}{\left(y_{t}-{\hat{y}}_{t}\right)}^{2}}$$ (6)

where *y*_*t*_ is the real stock index, ${\hat{y}}_{t}$ is the forecasted stock price, and *n* is the number of records.

Step 4 has six sub-steps, which is described the operation of the GA processes as follows:

Step4.1.1: Initialize the parameter for GA.

The initial population was set 80 individual solutions, and randomly generated in this sub-step, the SVR parameters are encoded into a chromosome by a binary string. In addition, the maximal generations, the crossover probability, the mutation probability, and are given as 2000, 0.8, and 0.08 [53] respectively.

Step4.1.2: Evaluate fitness.

This sub-step uses a pre-defined fitness function (RMSE) to evaluate fitness of each chromosome for determining the goodness of fit for each solution.

Step4.1.3: Check the stopping criterion

This sub-step sets the stopping rule: If one of the two conditions is got, then the GA process is stopped:

1. The maximal number of generations is reached (2000).
2. The optimal solution is smaller than the given minimal RMSE, the minimal RMSE is set as 10^−5^.

If the criterion is not achieved then repeatedly re-run a new iterative process (Step 4.1.2 to 4.1.5).

Step4.1.4: **Select the parents by the fitness function**

Selection is to screen out the fit chromosome to be copies for increasing the offspring sharing and eliminating the poorer chromosome for decreasing the offspring sharing. Roulette wheel selection is employed to select the chromosomes for reproduction.

Step4.1.5: **Perform crossover and mutation**

Re-unite the parents to produce and mutate offspring, this step one-point crossover is used. Then, it selected randomly a member of the population, and changed one randomly selected bit in its bit string representation [3].

Step5: Forecast the stock price by using the optimized models

From Step 4, the SVR parameters were determined. the testing data is applied to predict the next day’s stock prices by using the optimized forecast model.

Step6: Compare the performance

For evaluating the forecast accuracy, the propoesed model will be compared with the listing models under the RMSE criterion. The seven comparison models are as follows: (1) integrated KRR and SR (KRR-SR model), (2) integrated KRR and MARS (KRR-MARS model), (3) integrated KRR and GA-SVR (KRR-GA-SVR model), (4) integrated SR and MARS (SR-MARS model), (5) integrated SR and KRR (SR-KRR model), (6) integrated MARS and SR (MARS-SR model), and (7) integrated MARS and KRR (MARS-KRR model). Where KRR-SR model denotes using SR to build the forecast model after selecting the features by KRR, similarly other combined models have the same meanings, and the detailed abbreviations are presented in Table 3.

**Table 3:** The meaning of model abbreviation.

Abbreviation	Method
MARS	Multivariate adaptive regression splines
GA	Genetic algorithm
SR	Stepwise Regression
KRR	Kernel Ridge Regression
GA-SVR	Applying Genetic algorithm to optimize the Support vector regression parameters
KRR-SR	Employing KRR to select the key features and bulid model by SR
KRR-GA-SVR	Employing KRR to select the key features and bulid model by GA-SVR
KRR-MARS	Employing KRR to select the key features and bulid model by MARS
SR-KRR	Employing SR to select the key features and bulid model by KRR
SR-MARS	Employing SR to select the key features and bulid model by MARS
SR-GA-SVR (proposal model B)	Employing SR to select the key features and bulid model by GA-SVR
MARS-KRR	Employing MARS to choose the key features and bulid model by KRR
MARS-SR	Employing MARS to choose the key features and bulid model by SR
MARS-GA-SVR (proposal model A)	Employing MARS to choose the key features and bulid model by GA-SVR

## Experiment and comparisons

This study employs Taiwan’s stock as experimental datasets, the selected companies are different leading industries from “business today (www.businesstoday.com.tw)” which published the 1000 largest companies from Mainland China, Taiwan, and Hong Kong. The experimental datasets including Chunghwa Telecom (CHT), China Steel, Hon Hai, Cathay Financial Holdings and Taiwan Semiconductor Manufacturing Company (TSMC), were practically collected from 2003 to 2012. To compare the accuracy of a long test period forecast and short test period forecast, this study implements two experiments for each dataset, and the two experimental designs are listed in Table 4.

**Table 4:** The experiment of the long and short test period.

Experiment	Training period	Testing period
Short test period	2003 to 2011 year	2012
Long test period	2003 to 2009 year	2010 to 2012 year

First, this study conducts an initial experiment to explore the performance of the GA-SVR model. The forecasting performance of the GA-SVR is compared with SR, KRR, and MARS and the results are shown in Table 5. From Table 5, we can see that the GA-SVR model generates the smallest RMSE by the CHT, China Steel and Hon Hai datasets. Therefore, this study combines feature selection with the GA-SVR model as the proposed forecasting model. Second, this study sets the parameters of different forecasting models for the following experiment. In the parameter settings for the MARS model, the training data is utilized to build the MARS model, the maximal number of BFs of the MARS model’s parameter is set as 2000 and the other parameters are set as default [54]. For the KRR model, the parameter lambda for Tikhonov regularization of kernel ridge regression is set as 0.001 to build the forecasting model. In the SR model, the training data is employed to build the forecasting model, the high variance inflation factors (VIF) that are higher than 10 are removed first.

**Table 5:** The initial performance comparisons for three companies in RMSE.

company	model	Test period (year)	
		1	3
*CHT*	SR	0.0662	0.7664
	KRR	15.08679	34.3449785
	MARS	0.1435	2.3455
	GA-SVR	0.001162354	0.00329521
China Steel	SR	0.25348	0.34462
	KRR	3.91307785	4.16090037
	MARS	0.24370	0.32520
	GA-SVR	0.000651508	0.000922785
*Hon Hai*	SR	1.89469	2.20621
	KRR	4.27619926	12.5219447
	MARS	1.9228	2.151
	GA-SVR	0.002353294	0.006689822

Based on the initial experimental result (GA-SVR model performs better than the other models), this paper combines the GA-SVR model and feature selection method as the proposed model. Then, this study proposes model A and model B based on different feature selection methods. Model A uses MARS to select features, and model B utilizes stepwise regression as the feature selection method.

In comparison, this study selects the same number of features for different feature selection methods. The selected features by MARS, SR and KRR are listed in Table 6. After finding the key features, this study constructs the forecast model by SVR and optimizes the parameters of the MARS-GA-SVR and SR-GA-SVR models by GA, the optimized parameters are listed in Table 7.

**Table 6:** Selected features by MARS, SR and KRR for five companies.

Company	Method	Indicator1	Indicator2	Indicator3	Indicator4
*Chunghwa Telecom*	MARS	CDP value	MO2		
	SR	CDP value	MO1		
	KRR	Sale Month	P/E		
China Steel	MARS	Daily price change	TAIEX index	FBAQ	FBBQ
	SR	CDP value	MO2	MO1	-
	KRR	10BIAS	FBBQ	P/E	-
Hon Hai	MARS	FBBQ	ln(ROI)	Transaction volume	
	SR	EMA	MACD	ln(ROI)	
	KRR	Sale Month	RSI6	MO1	
Cathay Financial Holdings	MARS	ROI	FBBQ	CDP value	
	SR	5BIAS	MA5	MO2	
	KRR	ROI	TAIEX -Daily index change	TAIEX index	
TSMC	MARS	MO2	FBAQ	FBBQ	
	SR	CDP value	MO2	MO1	
	KRR	10W%R	MA15	EMA	

Note: FBAQ denotes The Final Best Ask Quote; FBBQ represents The Final Best Bid Quote

**Table 7:** The optimal parameters of GA searching for five companies.

Company	Method	Training period(year)	ε	C	*σ*	Training RMSE
Chunghwa Telecom	Proposed model A	9	0.2	85.115	621.018	0.10075
		7	0.2	85.115	621.018	0.08261
	Proposed model B	9	0.4	49.157	332.041	0.09963
		7	0.5	95.012	525.560	0.08395
	KRR-GA-SVR	9	0.1	94.601	687.127	1.11637
		7	0.2	96.738	988.762	1.10806
China Steel	Proposed model A	9	0.5	60.4713	0.0253	0.25387
		7	0.2	87.2495	9.356	0.20543
	Proposed model B	9	0.4	53.6446	6.1135	0.30688
		7	0.1	6.5271	0.6631	0.58843
	KRR-GA-SVR	9	0.5	51.7702	2.7261	0.504085
		7	0.3	25.0826	52.8511	0.31624
Hon Hai	Proposed model A	9	0.1	13.2041	134.089	0.26024
		7	0.4	74.9381	836.899	0.09486
	Proposed model B	9	0.3	17.8872	328.5681	0.11489
		7	0.5	67.8872	782.3546	0.09896
	KRR-GA-SVR	9	0.3	26.6437	44.2146	10.8103
		7	0.3	45.8883	20.0447	13.0136
Cathay Financial Holdings	Proposed model A	9	0.5	58.5778	3.428	0.97916
		7	0.1	60.9315	2.9168	1.04814
	Proposed model B	9	0.5	25.0216	0.834	1.04227
		7	0.2	37.1928	2.604	0.94704
	KRR-GA-SVR	9	0.2	15.4725	42.8472	1.69138
		7	0.3	5.9417	38.8681	1.29369
TSMC	Proposed model A	9	0.3	12.3139	4.3282	0.99827
		7	0.4	25.2229	3.4814	1.0201
	Proposed model B	9	0.3	72.1026	1.1468	0.98032
		7	0.5	26.0217	1.3375	0.98209
	KRR-GA-SVR	9	0.3	97.3413	731.4384	3.55611
		7	0.4	88.6254	48.0336	3.72557

### Experimental results

**Fig 2:**
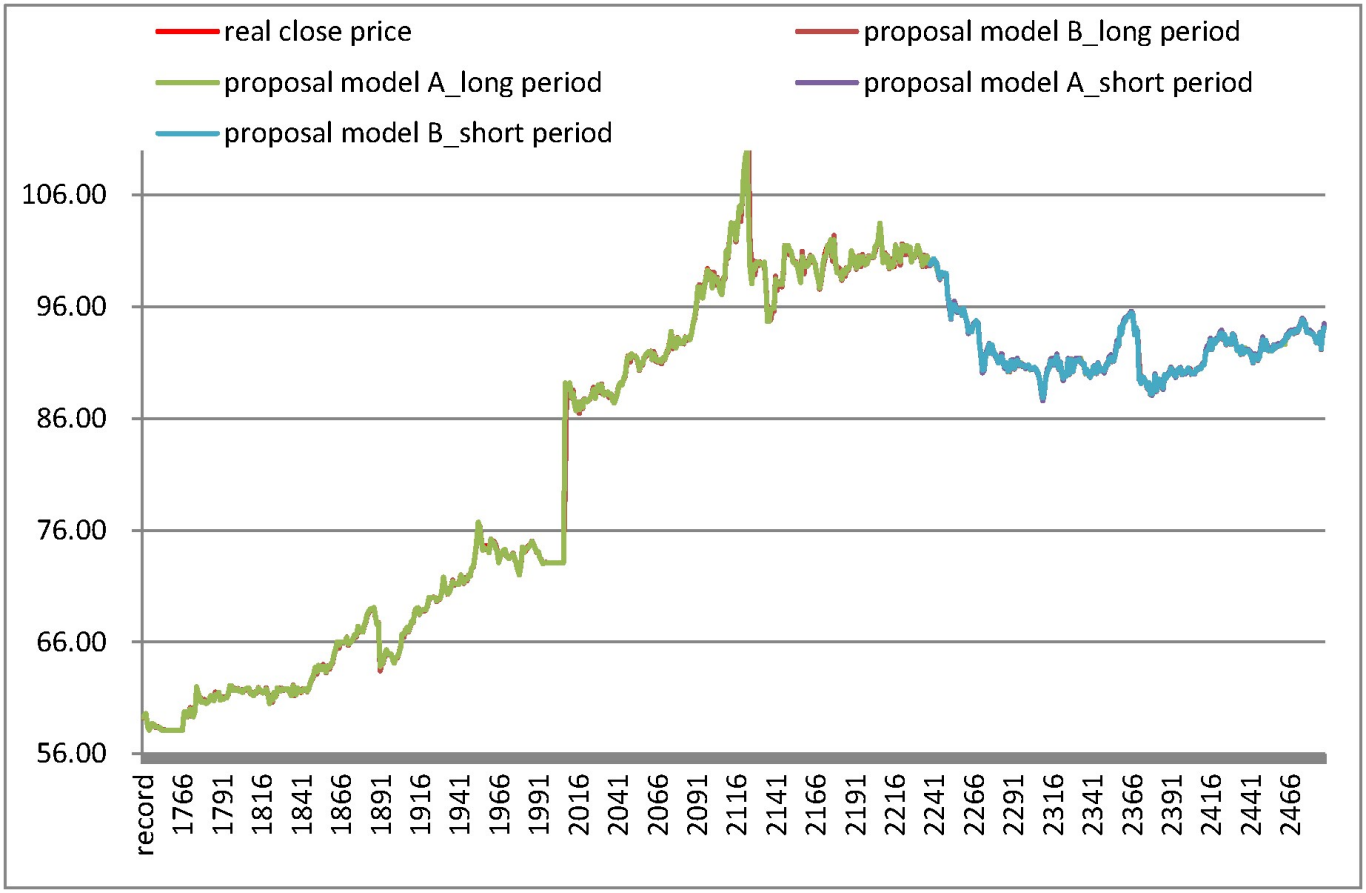
Results of forecasting short and long test period for Chunghwa Telecom datasets.

In the section, this study verifies the performance of the proposed model by using five different industry datasets including the Chunghwa Telecom datasets (CHT), China steel datasets, Hon Hai datasets, Cathay Financial Holdings datasets, and Taiwan Semiconductor Manufacturing Company datasets. The CHT datasets are employed in the first experiment; the computational process follows the proposed algorithm in Section 3. The predictions of the MARS-GA-SVR and SR-GA-SVR models are demonstrated in Fig 2. Fig 2 shows that the long test period results have more overlap between the forecast line and real closing price line than the results in the short test period in model A (MARS-GA-SVR). For proposed model B (SR-GA-SVR), there is more overlap between the forecast line and real close price line in the short test period results (in Fig 2). The RMSE for the listing models and proposed models, are shown in Table 8, and the results show that the performances of proposed models are better than other models. In the short test period, the proposed model B (SR-GA-SVR) generates the smallest RMSE in listing models as Table 8. Moreover, the proposed model A (MARS-GA-SVR) generates the smallest RMSE in the long test period (in Table 8).

**Table 8:** Performance comparisons for Chunghwa Telecom.

model	Test period(year)	
	1	3
KRR-SR	25.0596	28.4000
KRR-MARS	25.4612	37.0234
KRR-GA-SVR	2.5085	1.6955
MARS-SR	0.1527	0.1801
MARS-KRR	8.1484	27.6530
SR -MARS	0.1434	0.6356
SR-KRR	7.3393	75.7406
**Proposed model A**	0.0354	0.0586
**Proposed model B**	0.0342	0.0608

Proposed model A: Integrated MARS and GA-SVR model

Proposed model B: Integrated SR and GA-SVR model

**Fig 3:**
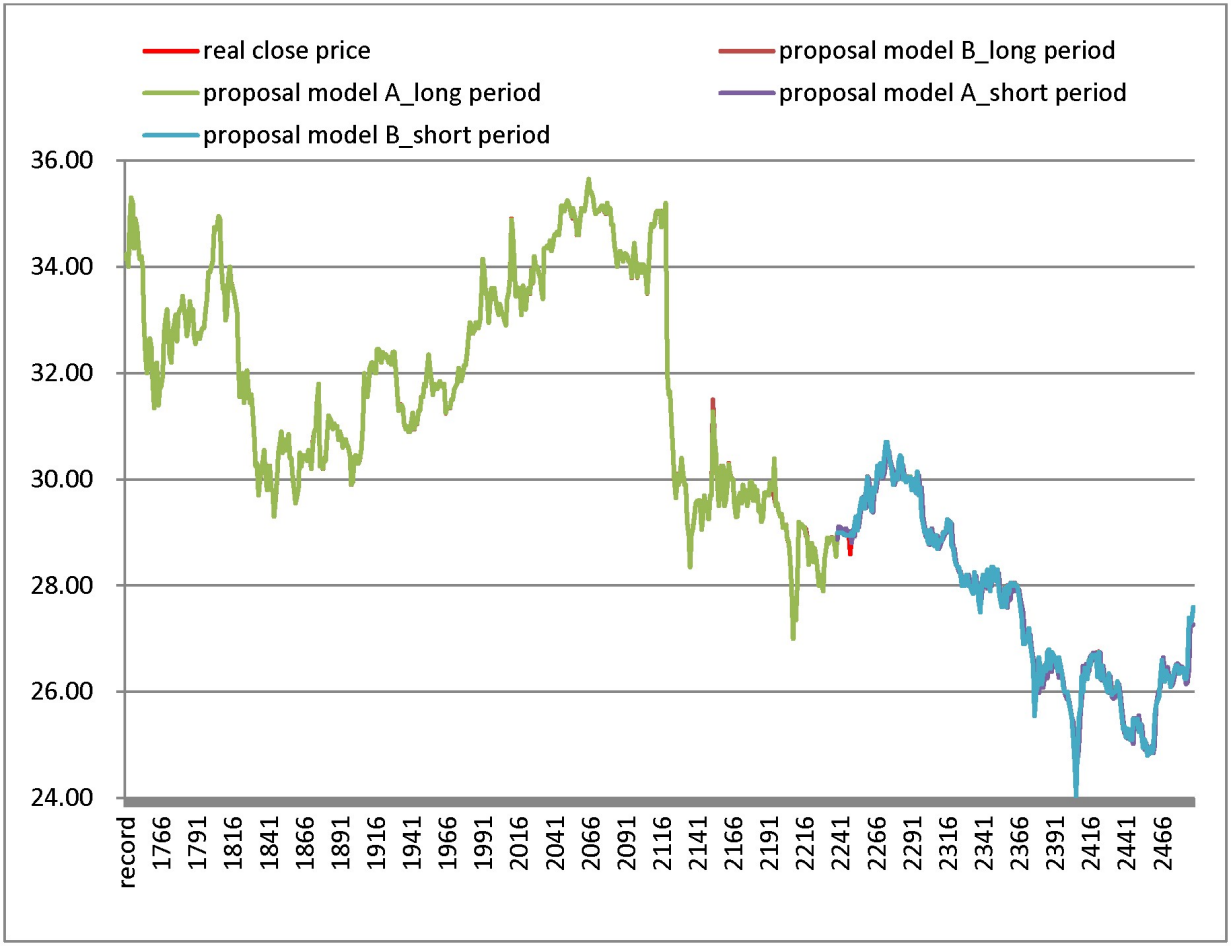
Results of forecasting short and long test period for China Steel datasets.

Table 9 and Fig 3 illustrate the numerical results for the China Steel datasets. From Fig 3, the result of model A shows that the real closing price line and the forecast line in the long test period have more overlap than in the short test period. Similarly, compared to the results in the long test period, the results generated by model B have more overlap between closing price line and the forecast line in short test period. From Table 9, the proposed models generate the smallest RMSE in the listing models. In the short test period, the proposed model B generates the smallest RMSE. In addition, the proposed model A generates the smallest RMSE in the long period.

**Table 9:** Performance comparisons for China Steel.

model	Test period(year)	
	1	3
KRR-SR	25.0596	28.4001
KRR-MARS	7.6083	0.3205
KRR-GA-SVR	1.634339328	1.01520238
MARS-SR	3.7657	3.9520
MARS-KRR	0.4634	0.4533
SR—MARS	0.2468	0.3250
SR—KRR	0.2452	0.3250
Proposed model A	0.2265	0.0381
Proposed model B	0.0370	0.3161

Proposed model A: Integrated MARS and GA-SVR model. Proposed model B: Integrated SR and GA-SVR model

**Fig 4:**
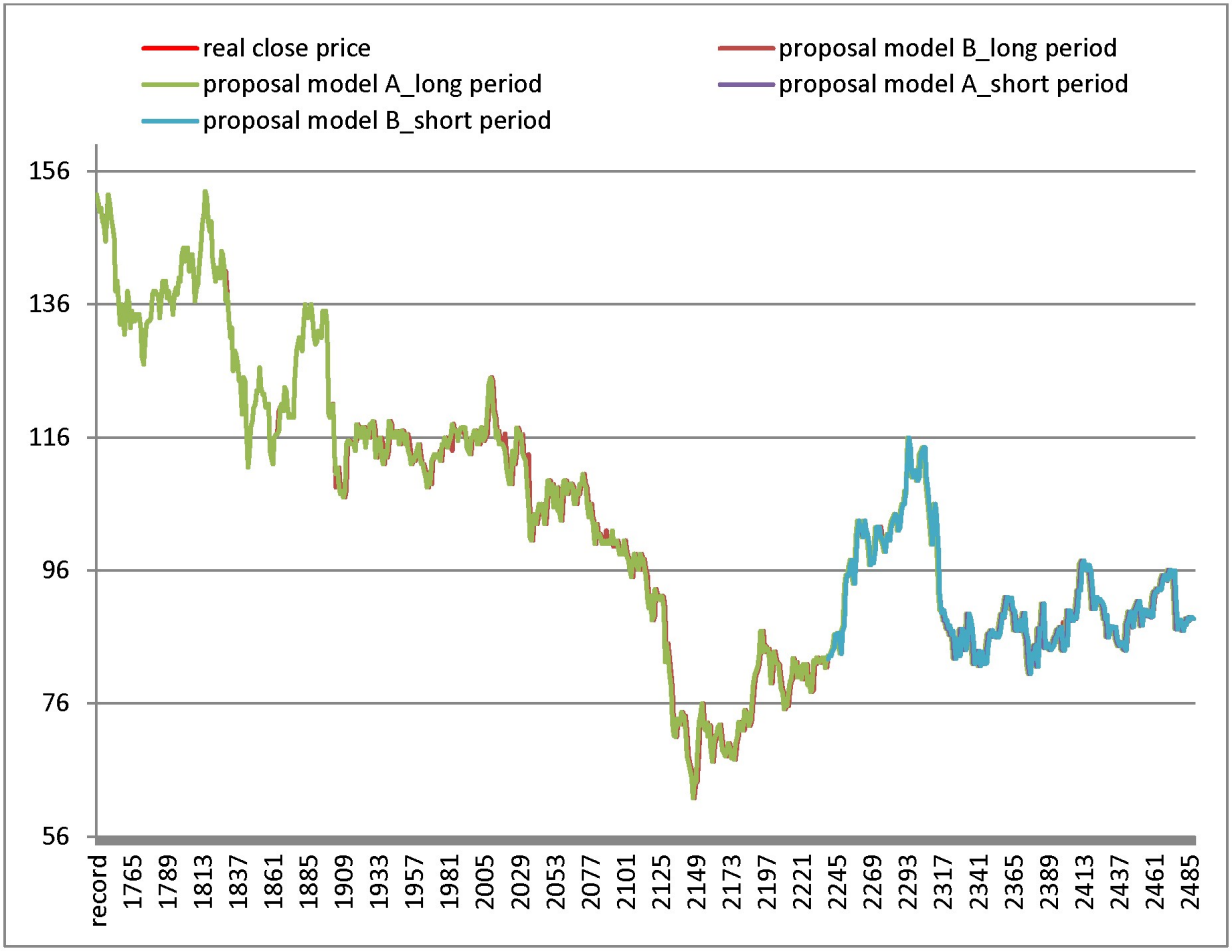
Results of forecasting short and long test period for Hon Hai datasets.

Experiments on the Hon Hai datasets are presented in Table 10 and Fig 4. Fig 4 shows the excellent performance of the proposed model (the forecast line almost completely overlaps the closing price line). Form Table 10, model A and model B generate the smallest RMSE in the long training period and the short training period, respectively.

**Table 10:** Performance comparisons for Hon Hai.

model	Test period(year)	
	1	3
KRR-SR	22.6070	76.1505
KRR-MARS	17.8506	339.1292
KRR-GA-SVR	0.4686	0.3136
MARS-SR	1.9738	2.1778
MARS-KRR	1.8837	2.2246
SR -MARS	1.8768	2.1328
SR -KRR	1.8528	2.1303
**Proposed model A**	0.0040	0.0087
**Proposed model B**	0.0030	0.0914

Proposed model A: Integrated MARS and GA-SVR model. Proposed model B: Integrated SR and GA-SVR model

**Fig 5:**
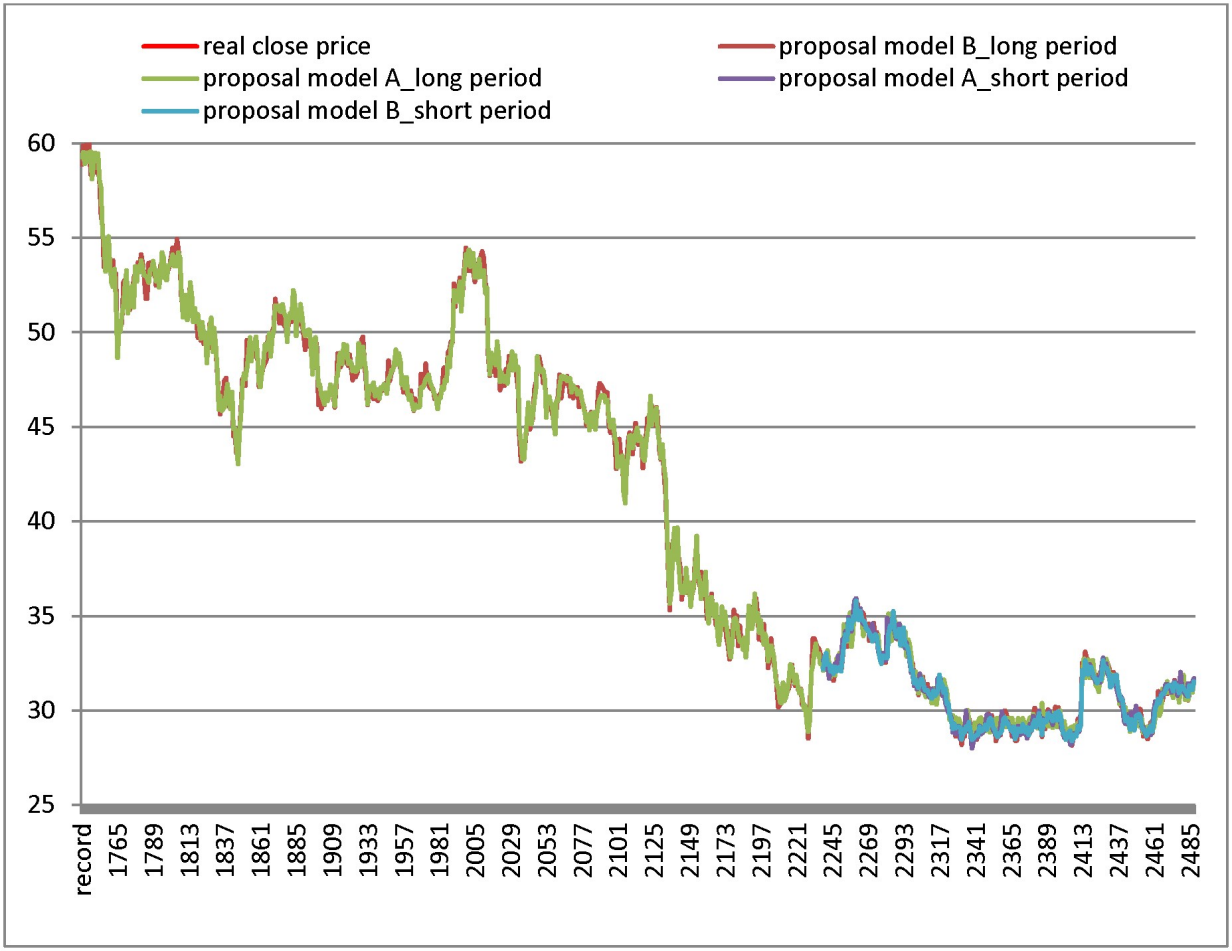
Results of forecasting short and long test period for Cathay Financial datasets.

Table 11 and Fig 5 show the experiments for the Cathay Financial Holdings datasets. In Fig 5, the numerical results clearly show that the forecast line deviates from the closing price line. From Table 11, the KRR-GA-SVR model generates the smallest RMSE in the short test period, and the proposed model B in the long test period generates a smaller RMSE than other models.

**Table 11:** Performance comparisons for Cathay Financial Holdings.

model	Test period (year)	
	1	3
KRR-SR	27.9581	30.3242
KRR-MARS	13.7496	24.5962
KRR-GA-SVR	0.0010	1.0663
MARS-SR	2.5218	0.8007
MARS-KRR	0.4663	0.7296
SR -MARS	0.4842	14.5722
SR -KRR	0.4831	0.7367
Proposed model A	0.2859	0.6552
Proposed model B	0.1671	0.5280

Proposed model A: Integrated MARS and GA-SVR model. Proposed model B: Integrated SR and GA-SVR model

**Fig 6:**
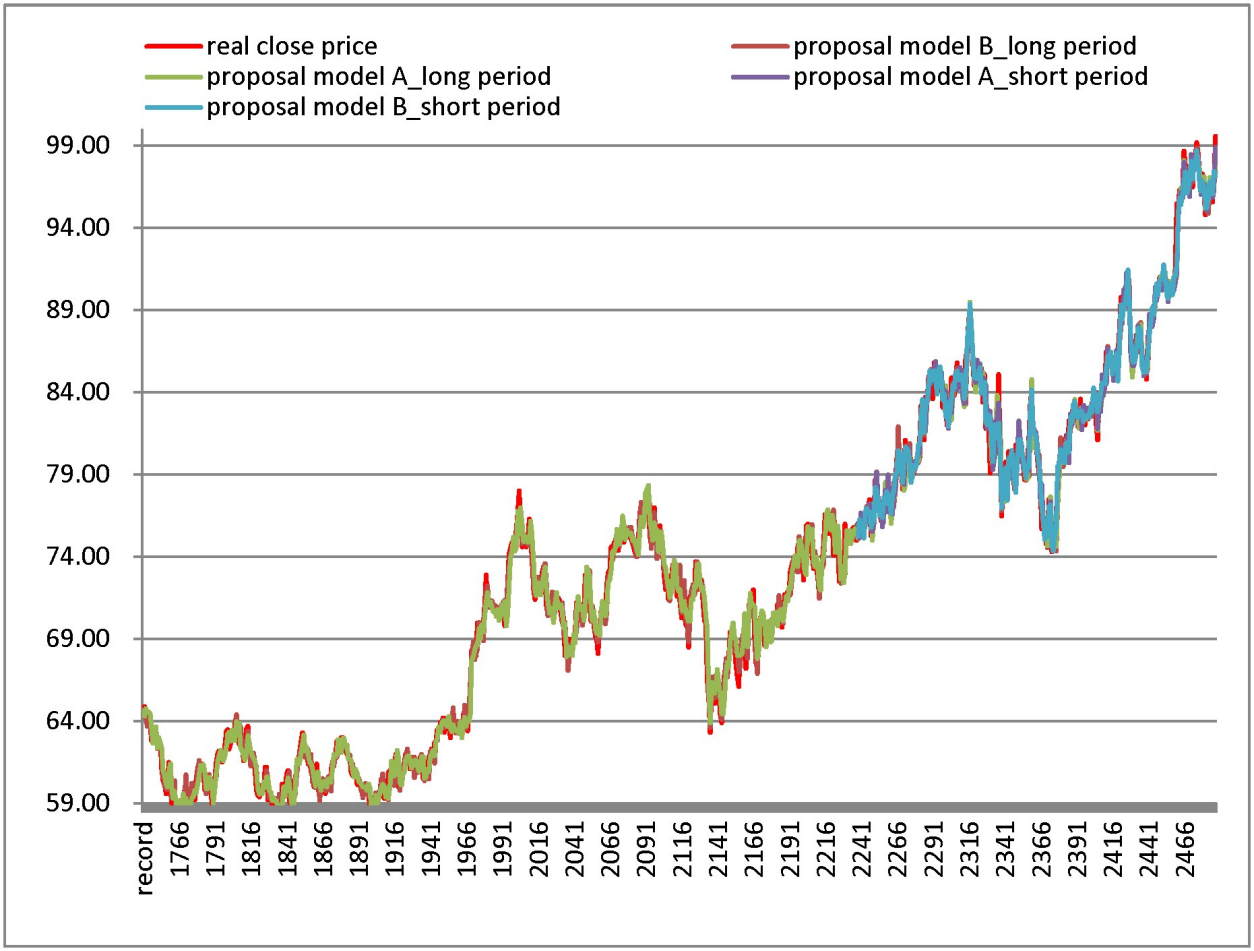
Results of forecasting short and test long period for TSMC datasets.

Next, experiments on the TSMC datasets are illustrated in Table 12 and Fig 6. The forecast results in Fig 6 show that the forecast line obviously deviates from the closing price line. From Table 12, the proposed model B generates the smallest RMSE in the short and long test periods.

**Table 12:** Performance comparisons for TSMC.

model	Test period(year)	
	1	3
KRR-SR	2.7440	12.3018
KRR-MARS	9.8224	125.9944
KRR-GA-SVR	5.0519	6.1786
MARS-SR	1.2980	0.9255
MARS-KRR	32.9380	29.0172
SR -MARS	1.2494	1.8027
SR—KRR	30.9380	22.4461
Proposed model A	0.8256	0.8395
Proposed model B	0.7292	0.7413

Proposed model A: Integrated MARS and GA-SVR model. Proposed model B: Integrated SR and GA-SVR model

### Significance test

To test whether proposed model is superior to the KRR-MARS, KRR-SR, KRR-GA-SVR, MARS-SR, MARS-KRR, SR-MARS and SR-KRR models in the stock price forecasting, this study applies the Wilcoxon signed-rank test. We use RMSE to test the significance between the proposed model and the listed models. Tables 13 and 14 present the Z statistic of the two-tailed Wilcoxon sign test between proposed models and the listed models.

**Table 13:** Wilcoxon sign test for different models comparison in short period.

model	KRR- MARS	KRR- SR	KRR- GA-SVR	MARS- SR	MARS- KRR	SR- MARS	SR- KRR	Proposed model B	Proposed model A
KRR- MARS	-	-0.135 (.893)	-2.023** (.043)	-2.023** (.043)	-0.674* (.5)	-2.023** (.043)	-0.674* (.5)	-2.023** (.043)	-2.023** (.043)
KRR- SR		-	-1.214 (.225)	-1.483 (.138)	-0.405 (.686)	-1.753* (.080)	-0.405 (.686)	-2.023** (.043)	-2.023** (.043)
KRR- GA-SVR			-	-0.135 (.893)	-1.483 (.138)	-0.944 (.345)	-1.214 (.225)	-1.753* (.08)	-1.753* (.08)
MARS- SR				-	-0.405 (.686)	-2.023** (.043)	-0.405 (.686)	-2.023** (.043)	-2.023** (.043)
MARS- KRR					-	-1.483 (.138)	-1.753* (.080)	-2.023** (.043)	-2.023** (.043)
SR- MARS						-	-0.405 (.686)	-2.023** (.043)	-2.023** (.043)
SR- KRR							-	-2.023** (.043)	-2.023** (.043)
Proposed model B								**-**	-0.674 (.5)

Note: The digital in parentheses is the corresponding p-value;

*:p<0.1;

**:p<0.05

**Table 14:** Wilcoxon sign test for different models comparison in long period.

model	KRR- MARS	KRR- SR	KRR- GA-SVR	MARS- SR	MARS- KRR	SR- MARS	SR- KRR	Proposed model B	Proposed model A
KRR- MARS	-	-1.214 (.225)	-1.753* (.08)	-1.753* (.08)	-1.753* (.08)	-1.753* (.08)	-0.944 (.345)	-2.023** (.043)	-2.023** (.043)
KRR- SR		-	-1.753* (.08)	-1.753* (0.08)	-0.944 (.345)	-1.753* (0.08)	-0.135 (.893)	-2.023** (.043)	-2.023** (.043)
KRR- GA-SVR			-	-0.135 (.893)	-1.214 (.225)	-0.135 (.893)	-1.214 (.225)	-2.023** (.043)	-2.023** (.043)
MARS- SR				-	-0.674 (0.5)	-0.674 (.5)	-0.405 (.686)	-2.023** (.043)	-2.023** (.043)
MARS- KRR					-	-1.214 (.225)	-0.405 (.686)	-2.023** (.043)	-2.023** (.043)
SR- MARS						-	-0.674 (.5)	-2.023** (.043)	-2.023** (.043)
SR- KRR							-	-2.023** (.043)	-2.023** (.043)
Proposed model B								-	-1.753* (.080)

Note: The digital in parentheses is the corresponding p-value;

*:p<0.1;

**:p<0.05

From Table 13, the proposed models have a significant difference (p<0.05) compared to other models in the short test period except for KRR-GA-SVR at the 0.05 significant level. Therefore, we can conclude that the proposed models are significantly better than KRR-MARS, KRR-SR, MARS-SR, MARS-KRR, SR-MARS, and SR-KRR models. However, we can see that there are no significant differences between proposed model A and B in the short testing period from Table 13. In the long testing period, the proposed models have a higher significance compared with the other models at the 0.05 significant level as shown in Table 14. Therefore, we can conclude that proposed model is significantly better than the listed models.

### Findings

Based on the experimental results, this study can summarize the findings as follows.

#### (1) Datasets quality

From Table 15, we find that of the five datasets with different fluctuations, the highest fluctuation range is the Hon Hai stock price. Despite the Hon Hai datasets having the highest fluctuation range, the proposed models can generate smaller RMSE than the listing models in the short and long test period as shown in Tables 16 and 17. Further, the China Steel stock price has the smallest fluctuation in the five datasets, and the proposed models still achieve better performance in both the short and long test periods, as shown in Tables 16 and 17. Finally, the results show that the two proposed models fit the forecast stock price for investors.

**Table 15:** The descriptive statistics for all datasets.

	TSMC	Cathay	Hon Hai	China Steel	CHT
Range	62.8	69.75	246	34.7	64
Minimum	36.8	24.05	54	19.2	46
Maximum	99.6	93.8	300	53.9	110
Mean	61.9997	53.6333	142.352	31.6591	66.9004
Std. Deviation	11.15911	14.83258	50.2475	6.48304	15.10203
Variance	142.526	220.006	2524.807	42.03	228.071

Note: Cathay denotes Cathay Financial Holdings

**Table 16:** The RMSE of all experiment for short testing period.

	TSMC	Cathay	Hon Hai	China Steel	CHT
KRR-SR	2.7440	27.9581	22.6070	25.0596	25.0596
KRR-MARS	9.8224	13.7496	17.8506	7.6083	25.4612
KRR-GA-SVR	5.0519	**0.0010**	0.4686	1.6343	2.5085
MARS-SR	1.2980	2.5218	1.9738	3.7657	0.1527
SR—MARS	1.2494	0.4842	1.8768	0.2468	0.1434
MARS-KRR	32.938	0.4663	1.8837	0.4634	8.1484
SR—KRR	30.938	0.4831	1.8528	0.2452	7.3393
Proposed model A	0.8256	0.2859	0.0040	0.2265	0.0354
Proposed model B	**0.7292**	0.1671	**0.0030**	**0.0370**	**0.0342**

Note: Cathay denotes Cathay Financial Holdings

**Table 17:** The RMSE of all experiments for long testing period.

	TSMC	Cathay	Hon Hai	China Steel	CHT
KRR-SR	12.3018	30.3242	76.1505	28.4001	28.4000
KRR-MARS	125.9944	24.5962	339.1292	0.3205	37.0234
KRR-GA-SVR	6.1786	1.0663	0.3136	1.0152	1.6955
MARS-SR	0.9255	0.8007	2.1778	3.9520	0.1801
SR—MARS	1.8027	14.5722	2.1328	0.3250	0.6356
MARS-KRR	29.0172	0.7296	2.2246	0.4533	27.6530
SR—KRR	22.4461	0.7367	2.1303	0.3250	75.7406
Proposed model A	0.8395	0.6552	**0.0087**	**0.0381**	**0.0586**
Proposed model B	**0.7413**	**0.5280**	0.0914	0.3161	0.0608

Note: Cathay denotes Cathay Financial Holdings

#### (2) Short and Long test period

**Fig 7:**
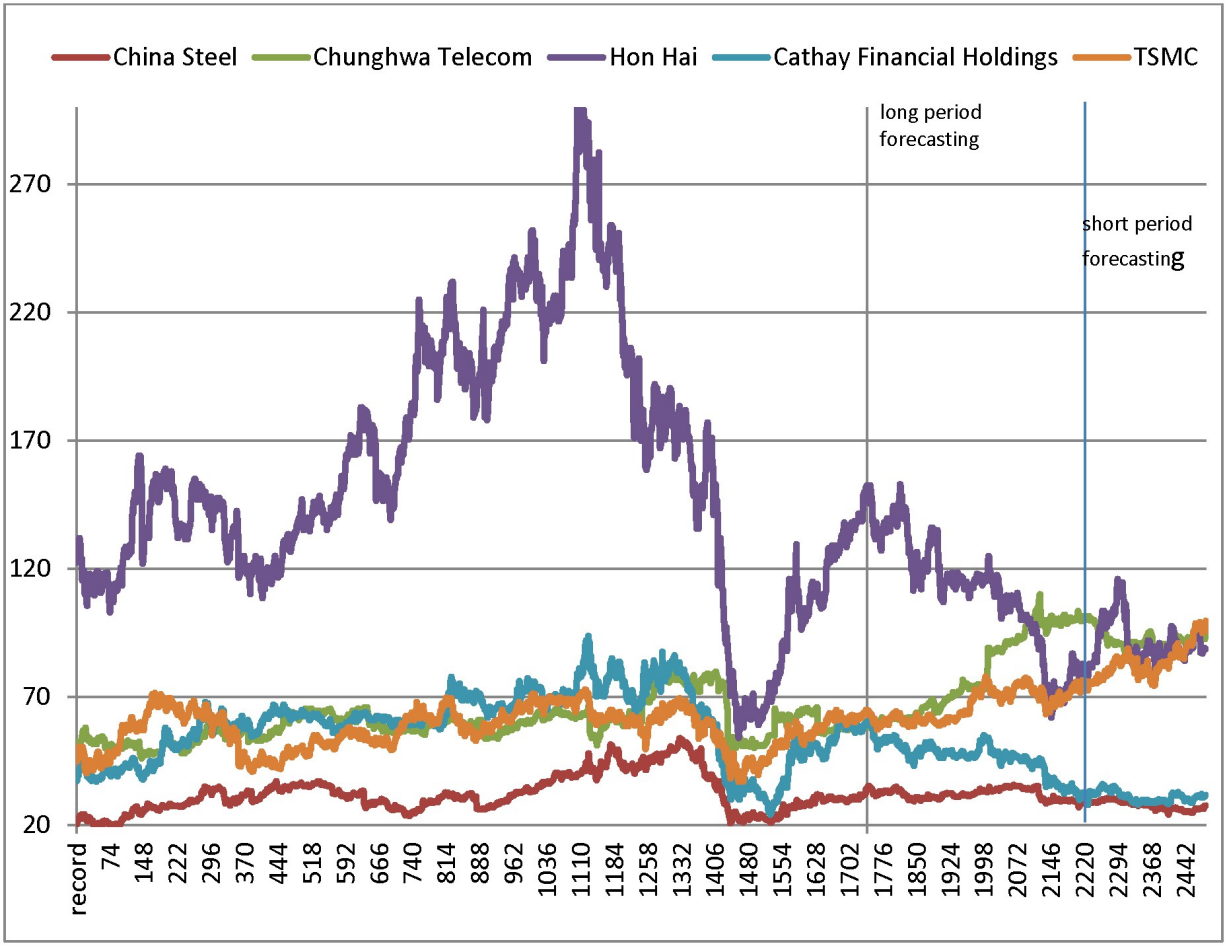
The closing prices of five companies from 2003 to 2012.

The experimental results of the forecasting models in the short and long test periods are listed in Tables 16 and 17, and we find that the accuracy of proposed models in the short test period is better than in the long test periods. From Fig 7, the figure shows that the stock indexes change dramatically in the long test periods; and the proposed models has better performance in larger price fluctuation. In the short test period, the results show that the proposed model B generates the smallest RMSE in the TSMC, Hon Hai, China Steel and CHT datasets, except Cathay dataset as Table 16. Because the fluctuation of Cathay price dataset in short period is smaller than other datasets as Fig 7. Therefore, we conclude that the proposed model B (SR-GA-SVR) has better performance than the listing models, especially in larger price fluctuation. *i*.*e*., we can confirm that the features selected by SR can effectively enhance the accuracy in the short testing period.

Similarly, in the long test period, from Table 17, the proposed model A (MARS-GA-SVR) has the smallest RMSE in the Hon Hai, China Steel and CHT datasets. Therefore, we conclude that MARS can also select better features for the proposed model when the stock price range changes dramatically.

#### (3) Selected feature

For the MARS selected features shown in Table 18, the feature “The Final Best Bid Quote” was chosen four times in five datasets and the forecasting results of proposed model A are better than the results of proposed model B in the long test period. Based on the reasons above, we can confirm that the feature “The Final Best Bid Quote” influences those stock prices forecasting in long testing period.

**Table 18:** The selected features for all datasets.

	Method	Indicator1	Indicator2	Indicator3	Indicator4
CHT	MARS	CDP value	MO2	-	-
	SR	**CDP value**	**MO1**	-	-
China Steel	MARS	Daily price change	**The Final Best Bid Quote**	The Final Best Ask Quote	TAIEX index
	SR	**CDP value**	**MO2**	**MO1**	-
Hon Hai	MARS	**The Final Best Bid Quote**	ln(ROI)	Transaction volume	-
	SR	EMA	MACD	ln(ROI)	-
Cathay Financial Holdings	MARS	ROI	**The Final Best Bid Quote**	CDP value	-
	SR	5BIAS	MA5	**MO2**	-
TSMC	MARS	MO2	The Final Best Ask Quote	**The Final Best Bid Quote**	-
	SR	**CDP value**	**MO2**	**MO1**	-

For the SR selected features as shown in Table 18, the features CDP, MO1 and MO2 are selected three times in five datasets. In addition, the proposed SR-GA-SVR shows with precise accuracy in the short testing period. Therefore, we find that the CDP, MO1 and MO2 have a great impact on forecasting stock prices for the short test period.

#### (4) Investor suggestion

After verifying the proposed models, this study can provide some suggestions to investors as references in the following:

- From Tables 16 and 17, the short test period forecasting is recommended, because it will be more accuracy than the long test period forecasting for investment stock.
- In the short period forecasting, we suggest using the proposed model B because it is more accuracy than proposed model A (see Table 16). Regarding key features as shown in Table 18, we suggest the investors consider the three key features: CDP, MO1 and MO2.
- In the long period forecasting, from Table 17, the proposed model A is recommended because it is more accuracy than proposed model B in the long testing period. From Table 18, the feature “The Final Best Bid Quote” should be considered as input variables in the long period forecasting.

## Conclusion

This study has proposed a new time-series model, which considers multifactor and reasonable selected key features into the GA-SVR model. The results show that proposed models can improve forecasting accuracy. Furthermore, the proposed models outperform the listed models in RMSE for Chunghwa Telecom, China Steel, Hon Hai, Cathay Financial Holdings and Taiwan Semiconductor Manufacturing Company datasets. In addition, from the findings and discussions, the proposed SR-GA-SVR outperforms the listing models in the short testing period, except Cathay Financial Holdings. Moreover, in the long testing period, the MARS-GA-SVR also has better performance. We find that the proposed model B almost has better performance than the listing models, especially in larger price fluctuation. i.e., the proposed model is more fit the dataset of larger price fluctuation. Finally, the research results can provide some suggestion to investors as references.

In future work, several issues from this study can be extended as follows:

1. Consider other features to train the model, such as company news, or government policies.
2. Apply the model to different application fields, such as electric loads and environmental pollution forecasting.
3. Employ other methods to improve proposed model, such as feature lags.

## Supporting information

S1 Dataset Five lead-industry-index. (ZIP)
